# Rigid iatrogenic hallux varus: a decades’ worth experience with arthrodesis of the metatarsophalangeal joint

**DOI:** 10.1007/s00264-024-06321-2

**Published:** 2024-09-19

**Authors:** S. Belfiore, S. Vaggi, F. Vitali, A. Zanirato, E. Quarto, M. Formica

**Affiliations:** 1 Ospedale Evangelico Internazionale - Salita Superiore, San Rocchino 31/A, 16122, Genova, GE Italy; 2 Dipartimento di Scienze Chirurgiche e Diagnostiche Integrate, Università degli studi di Genova - DISC, Viale Benedetto XV 6, 16132 Genova, GE Italy; 3 IRCCS Ospedale Policlinico San Martino – Clinica Ortopedica, Largo Rosanna Benzi, 10 16132 GENOVA, 16132 Genova, GE Italy

**Keywords:** Hallux varus, Hallux valgus failure, Hallux revision surgery, Hallux metatarsophalangeal arthrodesis, Intermetatarsal angle

## Abstract

**Purpose:**

Arthrodesis of the first ray metatarsophalangeal joint (MPJ) is the gold standard in iatrogenic hallux varus (IHV) in the presence of stiffness and osteoarthritis. The purpose of this study is to collect clinical and radiographic results and complications of MPJ arthrodesis in rigid iatrogenic HV.

**Methods:**

A retrospective evaluation of rigid iatrogenic HV undergoing arthrodesis with a minimum follow-up (FU) of two years was performed. The clinical parameters assessed were visual analog scale (VAS), the AOFAS Hallux Metatarsophalangeal-Interphalangeal Scale score and the satisfaction scale. The radiological parameters evaluated the first to second metatarsal angle (IMA) and the angle of hallux valgus (HVA). Complications were also analysed.

**Result:**

A total of 18 patients (19 procedures) with a mean FU of 5.5 ± 2.5 years were included. The mean VAS improved from 7.3 ± 1.6 to 1.3 ± 1.2 (*p* < 0.05) at the last FU. Similarly, the AOFAS Hallux Metatarsophalangeal-Interphalangeal scale score significantly improved to 82 ± 9.2 (*p* < 0.05). Radiological evaluation demonstrated a 1–2 IMA improvement from 4.4 ± 2.2° preoperatively to 8.9 ± 2.4° at 3 months post-operatively. Similarly, there was a significant (*p* < 0.05) improvement of the HVA from − 22.7 ± 4.1° to 13.1 ± 4.1° at three months post-operative (*p* < 0.05). No signification loss of correction was noted at the last follow-up (*p* > 0.05). In one case, a delayed fusion at the arthrodesis site required surgical revision to promote fusion. No patient experienced pain with stress from the first MTP joint arthrodesis site or identified the arthrodesis site as a source of pain. No patient required implant removal. Re-operation and revision rates were 5.3%. The overall complications rate was 15.8%.

**Conclusions:**

MPJ fusion effectively corrects Iatrogenic Hallux Varus in cases of rigid and fixed deformities in the medium- to long-term follow-up, with lasting improvements in AOFAS and VAS scores. The procedure is characterised by a non-negligible risk of complications, reoperations and revisions.

**Level of evidence:**

Level IV, case series.

**Supplementary Information:**

The online version contains supplementary material available at 10.1007/s00264-024-06321-2.

## Introduction

The hallux varus is a rare foot deformity compared to hallux valgus *(HV)*. Hallux varus is defined clinically as abduction or medial deviation of the big toe on the first metatarsal and radiologically as a negative metatarsophalangeal angle [1].

The tendency to varus results from an imbalance between the components that regulate the axis of the first metatarsophalangeal joint: bone, capsule-ligament structures, sesamoids and tendons of the flexor and extensor muscles of the big toe.

Several aetiologies have been recognised; nevertheless, the most common cause of hallux varus is iatrogenic. The literature reports an incidence of varus deformity from 2 to 15.4% following hallux valgus surgery [1, 2].

In iatrogenic hallux varus (IHV), the cause can be a bony imbalance related to previous osteotomy (excessive lateral displacement of metatarsal head, excessive resection of the medial prominence, sesamoidectomy) or a soft tissues imbalance (secondary to excessive lateral release, excessive retention of medial structures).

Imbalance in varus begins with minimal complaints, but often evolves into a rigid deformity with restriction of joint movement, arthritic alterations, contracture of the capsule-ligaments components and associated deformities such as metatarsophalangeal (MP) joint extension, interphalangeal joint flexion and proximal phalanx supination [1].

The patient experiences discomfort when wearing shoes, joint pain, postural alteration with walking in supination, lateral metatarsalgia [3]. In severe and chronic *IHV*, the big toe may not touch the ground due to dorsal capsule contracture of the MP joint and medially displaced extensor hallucis longus. The medially displaced tibial sesamoid may be palpated under the metatarsal head. Dorsal callosities on the interphalangeal area are also common due to the flexion contracture of the joint [1].

The treatment of iatrogenic hallux varus depends on the aetiology of the deformity (bony versus soft tissue imbalance) and the duration of the pathology. Early presentation and soft tissue imbalance aetiology of *IHV* can be treated non-operatively. Late presentation (more than 6–8 weeks), failure of the nonoperative management and aetiology of bone imbalance warrant surgical correction of the deformity [1].

The choice of the surgical procedures is based on deformity aetiology. Corrective procedures include medial capsular release, tendon transfer, tenodesis, ligamentoplasty, mini tightrope procedures, corrective osteotomies (of the first metatarsal and or proximal phalanx) and arthrodesis of the metatarsophalangeal joint [1, 4].

Preservation of MP joint mobility is the preferred approach; however, in the presence of degenerative changes and/or joint stiffness, arthrodesis is the treatment of choice [1, 5, 6].

Evidence in literature concerning arthrodesis of MP joint in IHV is scarce and based on studies with a small sample size. Clinical and radiological results are poorly reported. Only one study [7] described the radiographic results of MP arthrodesis for Hallux varus treatment. Furthermore, several complications of MP arthrodesis are reported in the literature, yet without a clear description of the incidence [1].

The present study aims to provide a detailed description of the clinical and radiographic results and complications of MP arthrodesis as a corrective strategy for rigid iatrogenic hallux varus deformity.

## Materials and methods

A single-centre retrospective study was conducted on patients undergoing surgery for iatrogenic hallux varus. Inclusion criteria were rigid IHV treated surgically with metatarsophalangeal arthrodesis; a minimum clinical and radiological FU of 24 months was established. Patients with MP bunion arthrodesis were excluded for any diagnosis other than hallux varus. Incomplete peri-operative and post-operative clinical and radiological data was another reason for exclusion from the analysis.

The hospital database was searched for MP arthrodesis for symptomatic and rigid IHV between 2011 and 2021.

All patients signed an informed consent form. The demographic data analysed were: gender, age and laterality of surgery.

### Surgical technique

All patients were treated with a standardised approach by a highly qualified foot surgeon (B.F.). A longitudinal lateral incision was made, centred on the MP big toe joint, and in cases where a previous incision was present, it was resumed. The joint was prepared by shaping the surfaces using a saw and Luer forceps. Bone stock preservation was the cornerstone of the surgical procedure; therefore, the saw cuts were as conservative as possible to create congruent surfaces of cancellous bone. The osteotomy of the first metatarsal was carefully planned preoperatively on a weight-bearing x-ray; the procedure was then performed under fluoroscopic control to ensure proper orientation and length. To optimise soft tissue preservation and avoid tissue tension, surgery was performed with a tourniquet. *The tourniquet helps to reduce surgical operative time*,* minimize blood loss*,* and improve visualization. Furthermore*,* the tourniquet prevents perioperative edema*,* thereby reducing tension on the skin suture. Before removing it*,* we apply compressive dressing to counteract the para-physiological edema.*

Arthrodesis was preferably achieved with two orthogonal shape memory staple configurations. Other fixation techniques were crossed screws, k-wires or plate.

The fusion was placed in slight anatomical valgus, with a 10–12° hallux valgus angle (HVA) to follow the common shape of the shoes and avoid compressions and pain. Similarly, we attempted to perform MP arthrodesis at approximately 20° of functional dorsiflexion to facilitate gait patterns and sporting activities. Adjunctive concomitant ipsilateral forefoot procedures were performed on a case by case basis. Postoperatively, patients were initially non-weight bearing for 6 weeks, using a postoperative shoe with a heel wedge to facilitate heel loading. Once healing was demonstrated on x-ray, they transitioned to protected weight-bearing at seven weeks, using comfortable shoes.

### Outcomes and complications

The radiological evaluation was performed before surgery, three months after surgery and at the last FU on AP radiographs of the foot in weight-bearing. The parameters analysed were the first to second metatarsal angle (IMA) and the angle of hallux valgus (HVA), as described by the Ad Hoc Committee of the American Orthopaedic Foot and Ankle Society on Angular Measurements [8].

All radiological measurements were performed by two different foot and ankle surgeons using the measuring instruments provided by the radiological software of the hospital.

The clinical parameters analysed were: American Orthopaedic Foot and Ankle Society (AOFAS) Hallux Metatarsophalangeal-Interphalangeal Scale [9], pain analog scale (VAS) [10] and a patient satisfaction scale. Clinical and radiological evaluation was performed before surgery, three months after surgery and at the last available FU.

Data were collected on systemic and local intraoperative and postoperative complications.

Each new surgery was considered as a reoperation; re-revisions instead included each new surgery on the first MP joint.

### Statistical analysis

Categorical variables were expressed as number of cases or percentage. Continuous variables were reported as mean ± standard deviation (SD) and range (minimum-maximum). Continuous data were analysed with a 2-tailed paired Student’s t test. For the analysis of radiological parameters, inter-observer variability was assessed with the K coefficient.

Statistical significance was set at *P* < 0.05.

## Results

A total of 19 first MP arthrodesis (18 patients; 17 women and 1 man) for rigid and symptomatic IHV were included in this analysis with a mean follow-up of 5.5 ± 2.5 years (range 2–11). *The mean interval between hallux valgus surgery and IHV surgery was 7.3 ± 6.3 years (range 1–26). The HV corrective surgery was: 11 Chevron osteotomies (57.9%) and eight minimally invasive distal metatarsal osteotomies (42.1%). Only 3 HV corrective surgery (15.7%) were performed in our centre; the other cases were treated in other hospitals.*

The mean age at the time of *IHV* surgery was 63 ± 7.8 years (range 45–75). The laterality was left in 53% of cases (10 pts), right in 42% (8 pts), and bilateral surgery was performed in one patient (5%). The arthrodesis site was fixed with two orthogonal shape memory staple (*LSM-Med – Republic of San Marino*) configurations in 15 cases (79%); in 3 cases (16%) with crossed screws, and a plate in another case (5%).

**Fig. 1:**
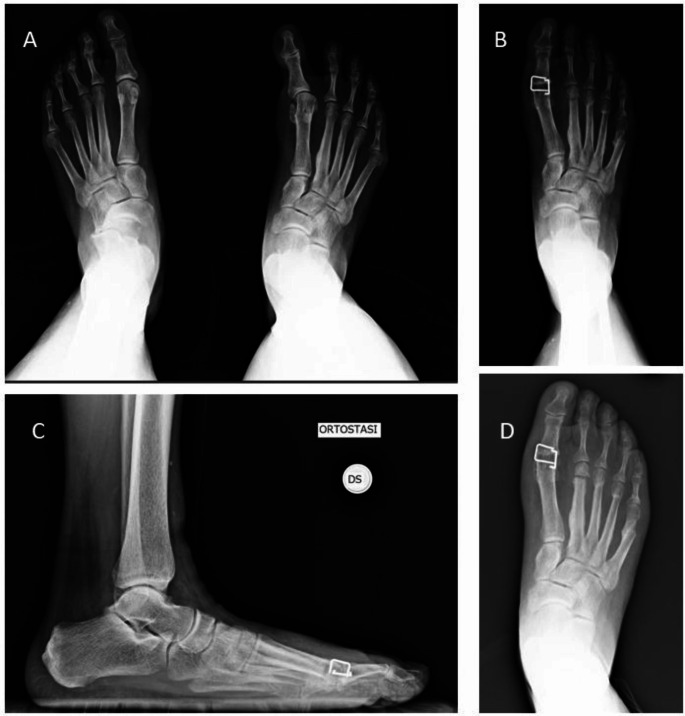
Illustrative case (A) Preoperative x-rays: Right iatrogenic hallux varus and transfer metatarsalgia; (B) 3-month follow-up x-rays: Hallux arthrodesis, Jimenez osteotomy on II-III-IV metatarsals with shortening, distal V metatarsal translation osteotomy for bunionette correction; C and D) 7-year follow-up x-rays: anteroposterior and lateral projection

In 15 patients (83.3%) concomitant procedures were performed on the ipsilateral forefoot: 12 patients underwent a dorsal wedge cut (Jimenez oblique V osteotomy ) on the lesser metatarsal for metatarsalgia, 11 patient underwent hammertoe correction with arthrodesis of the proximal interphalangeal or z plasty lengthening of extensor tendon, two patients underwent a bounionette correction with distal V metatarsal osteotomy, one Morton Neuroma excision, and one patient underwent lesser metatarsal head resection for concomitant rheumatoid arthritis (Lelievre technique). *For concomitant procedures K-wires fixation was used.* Figure 1 shows an illustrative case.

The average VAS improved from 7.3 ± 1.6 (range 3–9) to 1.5 ± 1.5 (range 0–5) points at three months after surgery (*p* < 0.05). At the last FU, the reported VAS score was 1.3 ± 1.2 (range 0–4) and no statistically significant differences were observed (*p* > 0.05). The mean AOFAS Hallux Metatarsophalangeal-Interphalangeal scale score was 30.2 ± 8.4 (range 13–42) points preoperatively; at 3 months a value of 81 ± 11.2 (range 65–100) was recorded (*p* < 0.05). At the last FU, the score was 82 ± 9.2 (range 70–100) (*p* > 0.05). Patients’ subjective satisfaction was reported as excellent in 10 feet (53%), good in 9 (47%) mainly for pain relief, correction of deformity and tolerance of shoe wear. All the patients stated that they would undergo the procedure again.

Radiological evaluation showed a 1–2 IMA improvement from 4.4 ± 2.2° (range 2–10) preoperatively to 8.9 ± 2.4° (range 4–15) three months after surgery. Furthermore, no statistically significant difference was detected at the last available FU (*p* > 0.05). Similarly, there was a significant improvement (*p* < 0.05) in the HVA from − 22.7 ± 4.1° (range − 30; -17) to 13.1 ± 2.1° (range 7–18) at three months post-operative, with no subsequent alteration (*p* > 0.05).

The inter-observer reliability of the radiological measurements was judged excellent according to the k coefficient of 0.86.

Wound dehiscence in the post-operative period was observed in two cases. These wound complications were resolved with targeted antibiotics and medications.

In one case, a delayed fusion was noted at the cross-screw arthrodesis site. In this patient, percutaneous drilling and secondary stabilization with k-wires was performed to promote fusion. Re-operation and revision rates were 5.3%. The overall complications rate was 15.8%.

No patient experienced pain with stress from the first MTP joint arthrodesis site or identified the arthrodesis site as a source of pain. No patient required implant removal at the last available FU.

Tables 1 and 2 reported demographic and surgical data, complication rate and clinical and radiological results.

**Table 1:** Demographic and surgical data. Complication rate. *HV: hallux valgus; IHV: iatrogenic hallux varus; MP: metatarsophalangeal*

*N*° of pts	18 (17 F; 1 M)
Mean age at HV surgery	56.1 ± 10.7 years (range 37–70)
HV corrective surgery	11 Chevron osteotomies (57.9%) 8 Minimally invasive distal metatarsal osteot. (42.1%)
Interval HV-IHV surgery	7.3 ± 6.3 years (range 1–26)
Mean age at IHV surgery	63 ± 7.8 years (range 45–75)
Mean FU	5.5 ± 2.5 years (range 2–11)
N° of MP arthrodesis	19
Fixation method	15 cases (79%): two orthogonal shape memory staples 3 cases (16%): two crossed screws, 1 case (5%): plate
Complications rate	15.8% 2 wound dehiscence 1 delayed fusion
Re-operation rate	5.3%
Revision rate	5.3%

**Table 2:** Clinical and radiological result. * statistically significant difference (*P* < 0.05)

	Preop	3 Months FU	Last FU
**VAS**	7.3 ± 1.6	1.5 ± 1.5 *	1.3 ± 1.2
**AOFAS**	30.2 ± 8.4	81.0 ± 11.2 *	82.0 ± 9.2
**IMA**	4.4 ± 2.2	8.9 ± 2.4	8.9 ± 2.1
**HVA**	-22.7 ± 4.1	13.1 ± 2.1	13.0 ± 2.4

## Discussion

The hallux MP arthrodesis technique for the treatment of hallux varus was discussed as early as 1989, when Mills and Menelaus [11] reviewed the results obtained after an average follow-up of 12.7 years. Arthrodesis is generally reserved for severe rigid deformity, failure of soft tissue surgery, or the development of osteoarthritis in the MP joint. Evidence in the literature concerning arthrodesis of MP joint in hallux varus is scarce and based on studies with a small sample size and limited analysis of clinical and radiological results and complications.

The aim of this study was to report clinical and radiological results and complication of arthrodesis in iatrogenic hallux varus. All patients included in the present study had a diagnosis of rigid and severe iatrogenic hallux varus. The study population analysed can therefore be considered homogeneous. To our knowledge, the present study presents results of the largest case series reported in the literature.

This study demonstrates that arthrodesis of the first MP joint is an effective treatment option in all cases of rigid and severe IHV, despite the different techniques used in the primary hallux valgus surgery, the severity of the deformity and the extent of residual bone stock.

Fusion of the first MTP joint, in our case series, was obtained in neutral rotation, with a 10–12° hallux valgus angle and approximately 20° of functional dorsiflexion. The literature suggested a position of the first MTP joint to achieve a stable fusion between 5° and 15° of valgus and between 20° and 25° of dorsiflexion with respect to the first metatarsal [12]. In our experience, the extent of dorsiflexion can be determined intraoperatively with a flat board to simulate the loading condition. The choice of the appropriate dorsiflexion angle is a cornerstone of the procedure. A lower dorsiflexion would increase pressure on the interphalangeal joint, leading to osteoarthritis at that level. Conversely, a greater angle would increase the pressure on the first metatarsal head, overloading the sesamoids and causing a claw deformity of the interphalangeal joint with severe difficulties with footwear [13].

Biomechanical studies in the literature indicate the dorsal plate and interfragmentary compression screw as the most stable fixation method to achieve fusion of hallux metatarsophalangeal joint [14]. In our experience, shape memory staples have proven to be effective for synthesis, affordable and less invasive than plates, reducing post-operative pain related to the hardware.

Considering the radiological parameters, the arthrodesis performed in the context of rigid IHV resulted in an increase of the 1st-2nd Intermetatarsal Angle (IMA) of 4.4 ± 2.4°. This implies that, while the Hallux Valgus Angle (HVA) was corrected during the procedure, the deforming forces, including those exerted by the abductor hallucis and flexor digitorum brevis, were repositioned in such a way that the 1–2 IMA was subsequently normalised. The correction obtained three months after surgery was then maintained at medium- to long-term follow-up demonstrating, a high rate of fusion obtained with the procedure.

Furthermore, the analysis of the preoperative clinical and functional data shows that iatrogenic hallux varus significantly results in severe discomfort when wearing shoes and during walking, joint pain and impairment of an individual’s quality of life. The main objective of arthrodesis is to alleviate pain symptoms and allow loading on the first ray. The level VAS at the last follow-up demonstrates the achievement of the first treatment goal. Similarly, AOFAS scores also demonstrate a significant and lasting functional improvement after surgery.

Grimes et al. [15] described that higher AOFAS scores are recorded in primary arthrodesis. Despite the lower AOFAS scores observed after a revision surgery. The fusion of the MP joint of the first ray appears to be a viable treatment option for hallux varus characterised by severe rigid deformity and development of MP osteoarthritis. Furthermore, when analysing the satisfaction rates reported by patients, 100% stating they would undergo the same procedure again. The great AOFAS improvement may be also related to a higher postoperative HVA, which is better adapted to most stocking designs. In addition, in our patient cohort we performed surgery to remove excess skin in the first interphalangeal space, which often causes discomfort. We recommend this procedure whenever a skin fold forms after hallux varus correction.

No significant complications were noted in the case series. In only one case, revision surgery was performed for delayed fusion of the first MP ray fusion. There were no cases of non-union in the last follow-up x-rays.

Pseudarthrosis, whether symptomatic or not, is one of the most relevant complications in arthrodesis of MP joint. In the literature, the fusion rate varies between 71% and 100% of cases depending on the procedure used, with no significant difference currently being demonstrated between the techniques used [16–18]. During a hallux arthrodesis, it is essential to adequately prepare the joint, as well as reduce and fix it. Non-union rates are influenced by the stability of the implant used. There is no consensus in the literature as to which fusion method is best, although several biomechanical studies have shown greater stability with the combination of dorsal plate and interfragmentary screw [19].

In our experience, shape memory staples have proven to be effective for synthesis, affordable, and less invasive than plates, reducing the risk of postoperative pain due to plate prominence. In our case series, delayed fusion was observed at the arthrodesis site fixed with crossed screws that required surgical revision to enhance fusion. Our preferred fixation technique (79%) of the fusion site was two orthogonal shape memory staple configurations. At the last available follow-up, no patients reported pain in the arthrodesis site.

The first MP joint fusion approach in IHV is a complex technique even for highly skilled foot surgeons, with a long learning curve. However, this technique can be mastered because the incidence of IHV ranges between 2% and 15.4% in the literature [20].

A limitation of the present analysis is the inclusion of patients undergoing concomitant corrective procedures; however, in foot surgery this element is common. Lastly, the study design is retrospective.

## Conclusions

MT fusion effectively corrects HVA in cases of stiff fixed deformities, leading to long-term improvements in AOFAS and VAS scores. The deformity correction achieved post-operatively is maintained at medium- to long-term follow-up. The procedure is characterised by a non-negligible risk of complications, reoperations and revisions.

## Electronic supplementary material

Below is the link to the electronic supplementary material.

Supplementary Material 1
