# Supplementary material for: Rigid iatrogenic hallux varus: a decades’ worth experience with arthrodesis of the metatarsophalangeal joint
Source: Int Orthop. 2024 Sep 19;48(11):2923–9. doi: 10.1007/s00264-024-06321-2 (PMC11490511; doi:10.1007/s00264-024-06321-2)
Supplement: Supplementary file 1 — Supplementary Material 1 [file 264_2024_6321_MOESM1_ESM.docx]

Dear Editors,

Here enclosed you will find a manuscript entitled “Rigid Iatrogenic Hallux Varus: a decades’ worth experience with arthrodesis of the metatarsophalangeal joint”.

Arthrodesis of the first ray metatarsophalangeal joint (MPJ) is the gold standard in iatrogenic hallux varus (IHV) in the presence of stiffness and osteoarthritis. Evidence in literature concerning arthrodesis of MP joint in IHV is scarce and based on studies with a small sample size. Clinical and radiological results are poorly reported.

The aim of this study was to report clinical and radiological results and complication of arthrodesis in iatrogenic hallux varus. To our knowledge, the present study presents results of the largest case series reported in the literature.

The authors declare no conflict of interests nor funding sources.

All authors contributed substantially to this work.

The study is original and the content of the article has not been published nor submitted for publication elsewhere.

We hope you will find our manuscript suitable for publication.

Yours sincerely,

On behalf of all authors
